# Enlarged perivascular spaces and cerebral small vessel disease

**DOI:** 10.1111/ijs.12054

**Published:** 2013-05-22

**Authors:** Gillian M Potter, Fergus N Doubal, Caroline A Jackson, Francesca M Chappell, Cathie L Sudlow, Martin S Dennis, Joanna M Wardlaw

**Affiliations:** Division of Clinical Neurosciences and SINAPSE Collaboration, University of Edinburgh, Western General HospitalEdinburgh, UK

**Keywords:** brain, cerebral infarction, leukoaraiosis, MRI, stroke, stroke subtypes

## Abstract

**Background and aims:**

Enlarged perivascular spaces (also known as Virchow–Robin spaces) on T2-weighted brain magnetic resonance imaging are common, but their etiology, and specificity to small vessel as opposed to general cerebrovascular disease or ageing, is unclear. We tested the association between enlarged perivascular spaces and ischemic stroke subtype, other markers of small vessel disease, and common vascular risk factors.

**Methods:**

We prospectively recruited patients with acute stroke, diagnosed and subtyped by a stroke physician using clinical features and brain magnetic resonance imaging. A neuroradiologist rated basal ganglia and centrum semiovale enlarged perivascular spaces on a five-point scale, white matter lesions, recent and old infarcts, and cerebral atrophy. We assessed associations between basal ganglia-, centrum semiovale- and total (combined basal ganglia and centrum semiovale) enlarged perivascular spaces, stroke subtype, white matter lesions, atrophy, and vascular risk factors.

**Results:**

Among 298 patients (mean age 68 years), after adjusting for vascular risk factors and white matter lesions, basal ganglia–enlarged perivascular spaces were associated with increasing age (*P* = 0·001), centrum semiovale–enlarged perivascular spaces (*P* < 0·001), cerebral atrophy (*P* = 0·03), and lacunar stroke subtype (*P* = 0·04). Centrum semiovale–enlarged perivascular spaces were associated mainly with basal ganglia–enlarged perivascular spaces. Total enlarged perivascular spaces were associated with increasing age (*P* = 0·01), deep white matter lesions (*P* = 0·005), and previous stroke (*P* = 0·006).

**Conclusions:**

Enlarged perivascular spaces are associated with age, lacunar stroke subtype and white matter lesions and should be considered as another magnetic resonance imaging marker of cerebral small vessel disease. Further evaluation of enlarged perivascular spaces in studies of ageing, stroke, and dementia is needed to determine their pathophysiological importance.

## Introduction

Perivascular spaces surround perforating arterioles and venules as they course from the subarachnoid space through the brain parenchyma (1). They are an important drainage conduit for cerebral interstitial fluid (2,3). When enlarged, these enlarged perivascular spaces (EPVS, also known as Virchow–Robin spaces) are commonly seen on T2-weighted brain magnetic resonance imaging (MRI) as punctate hyperintensities if perpendicular to, or linear if in the plane of, the image. However, although a few are normal at any age, large numbers are not (4). So although until recently regarded as normal, of no clinical consequence, and ignored in many studies of stroke, leukoaraiosis, and ageing, EPVS vary considerably in number between individuals and when numerous, are probably not normal. However, their precise associations are unclear.

Recent data show that EPVS increase in number with advancing age, either in older subjects (5,6), patients with mild stroke (7), patients with lacunar stroke (8), or patients with cerebral autosomal dominant arteriopathy with subcortical infarcts and leukoencephalopathy (9). EPVS were associated hypertension in older community-dwelling subjects (5), and in patients with mild (7) and lacunar (10) stroke. However, with the exception of Doubal *et al*. (7), none of the studies in lacunar stroke (8,10) included a relevant stroke control group with a nonlacunar stroke subtype and therefore are not able to say whether EPVS are associated specifically with small vessel disease as indicated by lacunar stroke. As EPVS follow the course of the penetrating arterioles, one might expect that they would be associated with cerebral small vessel disease (SVD), but this association might be difficult to distinguish from co-association with age or vascular risk as all increase with age. Unfortunately, only one previous study addressed the issue of whether EPVS were associated specifically with SVD by testing the association with lacunar versus nonlacunar stroke (7). White matter lesions (WMLs) are also a marker of SVD, and EPVS were associated with WML and lacunar (versus cortical) stroke (adjusting for age and vascular risk factors) (7), and with WML in a general older population (adjusting for hypertension, age, gender, and other vascular risk factors) (6), or only in patients with lacunar stroke (adjusted for several vascular risk factors) (8,10). EPVS were more frequent in patients with vascular versus Alzheimer's dementia (11), in subjects with worse cognition among otherwise normal older subjects (12), and among young patients with type 1 diabetes (13). They are also associated with active inflammation in multiple sclerosis plaques (14), and with several other nonvascular diseases such as myotonic dystrophy (15), Parkinson's disease (16), and depression at older ages (17).

These apparently varied associations make it unclear whether there really is a specific association between EPVS and SVD, as opposed to with general features of ageing or vascular risk factor exposure, or some other nonspecific factor. Despite increasing attention, data on EPVS, including their role in patients with ischemic stroke, and reliable information on their role in specific subtypes of stroke, are scant. We further examined associations between EPVS, WML, and stroke subtype in patients with different types of ischemic stroke, adjusting for vascular risk factors, to determine if there was a specific association between EPVS and SVD.

## Methods

### Study population

We included patients from a prospectively collected hospital-based stroke register of consecutive in- and outpatients with stroke seen at a large academic teaching hospital between April 2002 and May 2005 who underwent MRI (the Edinburgh Stroke Study; http://www.dcn.ed.ac.uk/ess/) (18). Patients who had only a computed tomography (CT) scan were not included. An experienced stroke physician diagnosed the stroke and determined the stroke subtype, using both clinical syndrome and brain imaging findings, according to the Oxford Community Stroke Project Classification: lacunar syndrome (LACS), partial and total anterior circulation syndromes (PACS, TACS, respectively), and posterior circulation syndrome (POCS) (19). We recorded patient demographics and risk factors including age, history of diabetes, history of hypertension, and previous clinical presentation with TIA or stroke (18). Hypertension and diabetes mellitus were defined as previous diagnosis of, or on current treatment for, hypertension or diabetes, respectively, according to standard clinical guidelines (20,21).

In the current analysis, we included only those patients with acute ischemic (first or recurrent) stroke who underwent MRI. We performed MRI when time from stroke onset was greater than five to seven-days or uncertain, if there was clinical uncertainty about the definite diagnosis of stroke or of the vascular territory involved (carotid or vertebrobasilar), if there was a potential underlying cause of stroke that required further investigation by MRI, if it was unclear whether the patient had had a recurrent stroke, or if the patient was suitable for inclusion into other studies of stroke requiring brain MRI.

The Edinburgh Stroke Study was approved by the Local Research Ethics Committee and all patients (or their relatives) gave written informed consent.

### MRI protocol and assessment

**Figure 1:**
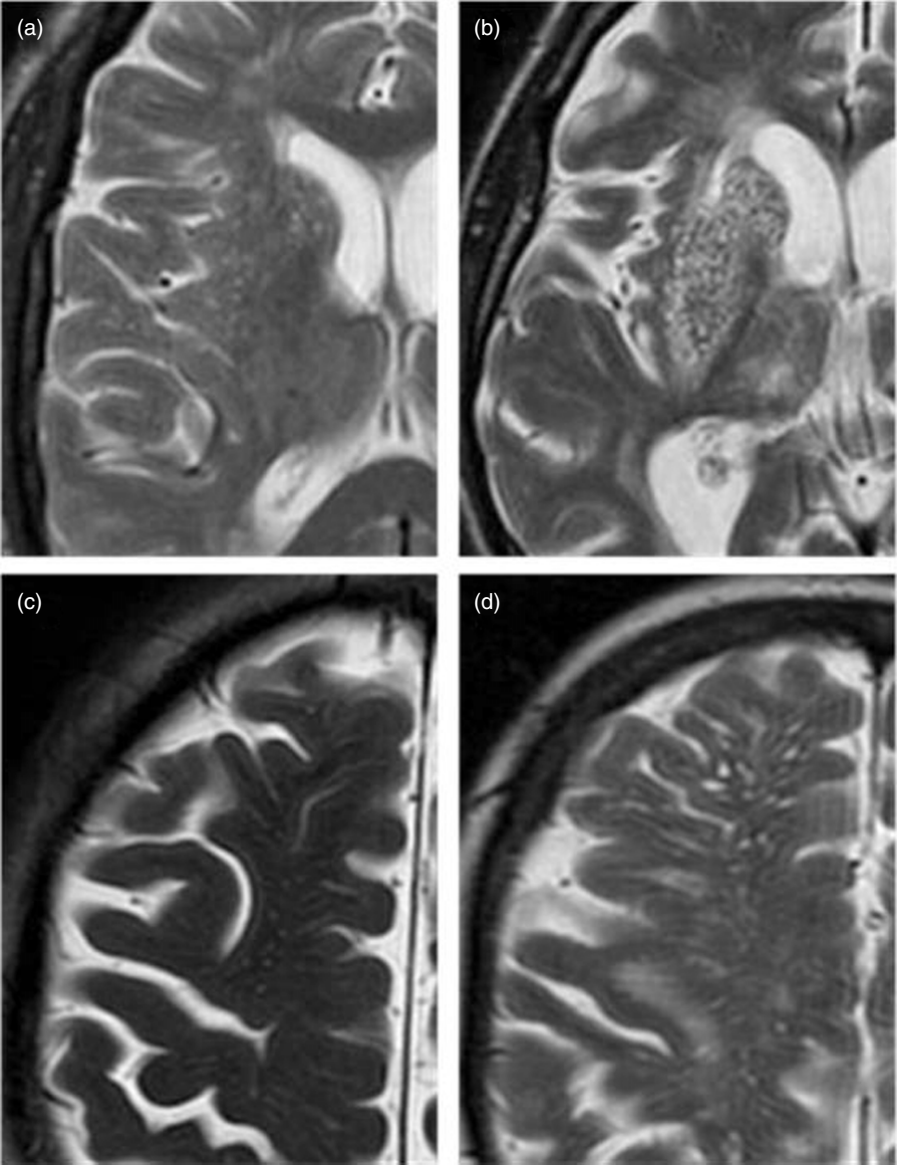
Magnified view of enlarged perivascular spaces (EPVS) in the basal ganglia (a, grade 2, 11–20 EPVS; b, grade 4, >40 EPVS) and centrum semiovale (c, grade 3, 21–40 EPVS; d, grade 4, >40 EPVS).

All patients had brain MRI on a 1·5T MR scanner (GE Signa LX EchoSpeed, Milwaukee, WI, USA) including the same T1- and T2-weighted, fluid attenuated inversion recovery (FLAIR) and T2* sequences, and diffusion-weighted imaging (DWI). A neuroradiologist (GMP) reviewed the images, blinded to clinical details, and recorded the location and size of recent infarcts. We defined: recent infarcts as hyperintense on DWI, hypointense on the apparent diffusion coefficient map, and either normal or hyperintense to normal brain on FLAIR/T2 imaging (less hyperintense than cerebrospinal fluid on T2); acute lacunar infarcts as round or ovoid lesions measuring ≤20 mm in maximal diameter in the white matter, basal ganglia (BG), or brainstem; EPVS as ≤2 mm round or linear cerebrospinal fluid-isointense lesions (T2-hyperintense and T1/FLAIR-hypointense with respect to brain) along the course of penetrating arteries. We rated EPVS on T2 imaging (with all sequences available) in the BG and centrum semiovale (CS) as 0 = none, 1 = 1–10, 2 = 11–20, 3 = 21–40, and 4 = >40 EPVS per side (7) using the worse side if there was any asymmetry (Fig. 1). We calculated total EPVS scores (0–8) by summing the CS- and BG-EPVS scores. We rated periventricular and deep WML as 0–3 with the Fazekas method (22). We recorded presence of old infarcts (using all sequences) and rated deep and superficial cerebral atrophy from 0 to 6 using a validated scale, where 0 = none and 6 = severe (23).

### Statistical analysis

We performed univariate and multivariate analyses to determine associations between BG- and CS-EPVS and explanatory variables (age, clinical history of previous stroke, hypertension, diabetes, periventricular WML, deep WML, old infarcts, atrophy and lacunar versus other stroke subtypes) in the BG and CS. BG- and CS-EPVS were not normally distributed and, to permit binary logistic regression, we dichotomized BG- and CS-EPVS into 0 (EPVS scores 0, 1) and 1 (EPVS scores 2, 3, 4), reflecting mild versus moderate to severe EPVS, as used previously (8). Total EPVS was normally distributed. We assessed univariate associations of total EPVS (using the Pearson correlation, Student's *t*-test, and linear regression) then used multiple linear regression to determine independent associations of total EPVS with vascular risk factors, lacunar stroke subtype, and MRI markers of cerebral SVD). We dichotomized scores for WML (0–1 versus 2–3) and for atrophy (combining superficial and deep atrophy scores to give a score 0–6, then dichotomizing as 0–3 versus 4–6). We dichotomized stroke subtype into lacunar and nonlacunar stroke; patients with PACS, TACS, and POCS were classified as nonlacunar stroke. The alpha value for significance was set at 0·05. Minitab Statistical Software (Version 15, Minitab Inc., State College, PA, USA) was used for all analyses.

## Results

Among the 1311 acute ischemic stroke patients recruited to the Edinburgh Stroke Study, 313 underwent brain MRI. T2 imaging was of insufficient quality to rate EPVS in 17 patients. Therefore, we included 298 patients of mean age of 68 (±13) years, of whom there were 89 (30%) with LACS and 209 (70%) with other subtypes: 113 (54%) with PACS, 20 (10%) with TACS, 69 (33%) with POCS, and 7 in whom the stroke subtype was uncertain (3%) (Table 1). Mean total EPVS score was 4, and the median score for both periventricular and deep WML was 1; moderate to severe atrophy was present in 61 (21%) patients (Table 1). Patients undergoing MRI were slightly younger when compared with the 1311 ischemic stroke patients from which we identified our study population, with a higher proportion of males and a lower prevalence of diabetes, however with a similar proportion of PACS and LACS; only a proportion of patients with previous stroke was significantly different between the two groups, higher in those undergoing MRI (*P* = 0·02; Supporting Information Table S1, online only).

**Table 1:** Baseline characteristics of all subjects (*n* = 298)

Characteristic	
Mean age in years (SD)	68 (13)
Previous TIA *n* (%)	51 (17)
Previous stroke *n* (%)	79 (27)
Hypertension *n* (%)	156 (52)
Diabetes *n* (%)	27 (9)
Lacunar stroke subtype *n* (%)	89 (30)
Deep WML Fazekas score, median (IQR)	1 (1–2)
Periventricular WML Fazekas score, median (IQR)	2 (1–2)
Atrophy *n* (%)	61 (21)
Old infarcts *n* (%)	89 (30)
Mean total EPVS score (SD)	4 (2)

EPVS, enlarged perivascular spaces; IQR, interquartile range; SD, standard deviation; TIA, transient ischemic attack; WML, white matter lesions.

### Associations of BG EPVS

In univariate analyses, BG-EPVS were associated with age, periventricular WML, deep WML, CS-EPVS, and atrophy (all *P* < 0·001, Table 2). In multivariable analysis, only age (*P* = 0·001), CS-EPVS (*P* < 0·001), atrophy (*P* = 0·03), and lacunar stroke subtype (*P* = 0·04) remained significant (Table 2).

**Table 2:** Univariate and multivariate* associations for dichotomized basal ganglia and centrum semiovale EPVS (*n* = 298)

Variable	Dichotomized basal ganglia EPVS‡				Dichotomized centrum semiovale EPVS‡			
	Univariate OR (95% CI)	Univariate *P* value	Multivariate OR (95% CI)	Multivariate *P* value	Univariate OR (95% CI)	Univariate *P* value	Multivariate OR (95% CI)	Multivariate *P* value
Age	1·08 (1·05–1·10)†	<0·002	1·06 (1·02–1·09)†	0·001	1·03 (1·01–1·05)†	0·002	1·00 (0·98–1·03)	0·76
Hypertension	1·78 (1·12–2·82)†	0·015	0·94 (0·50–1·76)	0·84	1·5 (0·89–2·52)	0·13	1·13 (0·63–2·01)	0·69
Diabetes	0·65 (0·29–1·46)	0·3	0·68 (0·24–1·98)	0·48	1·27 (0·49–3·28)	0·62	1·58 (0·56–4·46)	0·39
Deep WML	3·00 (2·19–4·11)†	<0·001	1·43 (0·81–2·54)	0·22	1·29 (0·98–1·7)	0·07	0·75 (0·44–1·29)	0·29
Periventricular WML	3·48 (2·53–4·80)†	<0·001	1·73 (0·95–3·15)	0·08	1·46 (1·1–1·93)†	0·01	1·11 (0·63–1·97)	0·72
Old infarcts	1·59 (1·00–2·54)	1·05	0·76 (0·40–1·45)	0·4	1·32 (0·77–2·24)	0·31	1·22 (0·66–2·23)	0·53
BG EPVS	NA	NA	NA	NA	2·56 (1·76–3·74)†	<0·002	2·96 (1·87–4·69)	<0·001
CS EPVS	3·00 (2·21–4·07)†	<0·001	3·27 (2·20–4·85)†	<0·002	NA	NA	NA	NA
Atrophy	3·64 (1·95–6·81)†	<0·001	2·72 (1·08–6·84)†	0·03	1·01 (0·53–1·91)	0·98	0·55 (0·24–1·30)	0·17
Lacunar stroke subtype (vs. non-lacunar)	1·36 (0·79–2·34)	1·26	2·08 (1·04–4·17)†	0·04	0·8 (0·44–1·46)	0·47	0·69 (0·37–1·29)	0·25

* All variables included in multivariate analysis, except for CS or BG as shown.

† Significant at *P* < 0·05.

‡ Dichotomized into 0 (EPVS scores 0, 1) and 1 (EPVS scores 2, 3, 4), reflecting mild versus moderate to severe EPVS.

BG, basal ganglia; CI, confidence interval; CS, centrum semiovale; EPVS, enlarged perivascular spaces; NA, not applicable; OR, odds ratio per unit increase in explanatory variable; WML, white matter lesions.

### Associations of CS EPVS

In univariate analyses, CS-EPVS were associated with age (*P* = 0·002), periventricular WML (*P* = 0·01), and BG-EPVS (*P* = <0·001). However, only BG-EPVS was significantly associated with CS-EPVS in multivariate analysis (*P* = <0·001), after adjusting for all other explanatory variables (Table 2).

### Associations of total EPVS

In univariate analyses, total EPVS were associated with increasing age, hypertension, periventricular WML and deep WML (all *P* < 0·001), atrophy (*P* = 0·001), and old infarcts (*P* = 0·04; Table 3). After adjustment for vascular risk factors, stroke subtype, and periventricular WML, only age (*P* = 0·01), deep WML (*P* = 0·005), and history of previous stroke (*P* = 0·006) were statistically significantly and independently associated with total EPVS (Table 3).

**Table 3:** Univariate and multivariate* associations with total (basal ganglia and centrum semiovale) EPVS (*n* = 298)

Variable	Univariate		Multivariate	
	Statistic used and test score	*P* value	Beta coefficient	*P* value
Age	Pearson coefficient 0·422	<0·001†	0·02	0·01†
Previous stroke	*t*-test difference in EPVS score 0·033 (−0·393 to 0·460)	0·88	−0·27	0·006†
Hypertension	*t*-test difference in EPVS score −0·676 (−1·049 to −0·302)	<0·001†	0·25	0·15
Diabetes	*t*-test difference in EPVS score 0·356 (−0·294 to 1·005)	0·27	−0·31	0·31
Periventricular WML	Linear regression adj *r* squared 24·8%	<0·001†	0·31	0·08
Deep WML	Linear regression adj *r* squared 22·6%	<0·001†	0·44	0·005†
Atrophy	Linear regression adj *r* squared 3·4%	0·001†	−0·003	0·42
Old infarcts	*t*-test difference in EPVS score 0·4 (0·02–0·779)	0·04†	−0·01	0·97
Lacunar stroke subtype (versus nonlacunar)	*t*-test difference in EPVS score 0·16	0·48	0·15	0·42

EPVS, enlarged perivascular spaces; WML, white matter lesion.

* All variables included in multivariate analysis.

† Significant at *P* < 0·05.

## Discussion

Among 298 patients with ischemic stroke, we demonstrate a consistent association between EPVS and SVD in the form of lacunar stroke and WML, after adjusting for age and vascular risk factors. Total EPVS were associated with increasing age, deep WML, and previous stroke; BG and CS-EPVS are closely co-associated; and BG-EPVS were associated with increasing age, lacunar stroke, and atrophy, after adjusting for potential covariates. Although similar associations have been assessed previously, only one previous study compared EPVS associations between lacunar and cortical stroke (7), and our study is the first to specifically assess the association between EPVS and lacunar stroke among patients with all types of ischemic stroke. Thus, while EPVS undoubtedly are a marker of age, they are also specifically associated with SVD as evidenced by lacunar stroke and WML. Neither are they just a marker of vascular risk factor exposure nor stroke in general. The stronger association of lacunar stroke subtype with BG- than with CS-EPVS is interesting and requires further evaluation as it may be relevant to mechanisms of damage to the perforating arterioles that cause lacunar stroke. It might also suggest that it is only necessary to quantify BG-EPVS.

The strengths of our study include the use of carefully standardized research methods, subtyping by an experienced stroke physician, the inclusion of different stroke subtypes, and the reasonable sample size, the second largest among all EPVS studies and the largest in stroke patients to date. The EPVS rating was performed without knowledge of the hypothesis being tested, avoiding expectation bias. EPVS were assessed in the two sites where they are most commonly found. We used a rating scale previously shown to have high intrarater agreement (12) and which permits a full range of EPVS to be recorded.

The limitations of our study include the use of a single brain imaging rater. However, ‘recalibration’ using a test set of images was performed during rating in order to try to minimize intrarater variability. Loss of power may have occurred due to categorizing rather than precisely counting the number of EPVS; however, counting would take a very long time and categorization works well in other situations, for example, for WML rating. Overall, 23% of patients in the Edinburgh Stroke Study underwent MRI, which may have introduced a selection bias for stroke severities but is unlikely to change the results of the primary analysis of lacunar versus nonlacunar stroke; the group undergoing MRI had similar proportions of PACS and LACS to the whole registry cohort, and LACS were not overrepresented (at 30%) as in at least three other studies [50% (7) and 100 (8,10)]. The minor differences between patients undergoing MRI and those that did not (slightly younger, more males, fewer with vascular risk factors) are unlikely to have influenced our results. Our definition of LACS included only anterior circulation lacunar infarcts. Lacunar infarcts in the posterior circulation, as well as posterior circulation cortical infarcts, were included in our definition of POCS. However, only a very few patients had brainstem lacunar infarcts; we did not quantify brainstem EPVS and therefore we felt justified in including the POCS in the nonlacunar subtype for the present analysis, which, if anything, is likely to have diluted rather than falsely inflated the association with lacunar subtype. Hypertension is often underdiagnosed and undertreated; therefore, our reliance on prior diagnosis of, or treatment for, hypertension may have influenced our assessment, and could partly account for the lack of association in our study.

Our finding of an association between BG-EPVS and WML is similar to findings by Doubal *et al*. (7) in patients with lacunar versus mild cortical stroke, and by Ohba *et al*. in patients just with lacunar stroke (8), using the same method of EPVS assessment; with Zhu *et al*. (6), whose image assessment differed for EPVS, WML, and atrophy, and who studied an elderly population rather than patients with stroke; and with Rouhl *et al*. (10) who found associations between BG-EPVS and age, hypertension, asymptomatic lacunar infarcts, and WML, just in patients with lacunar stroke. The association between EPVS and microbleeds – a further likely marker of cerebral SVD – was not assessed in this study; however, it merits attention in future studies. Associations between EPVS and cognitive function were not assessed as cognitive data were not recorded, but may may also be worthy of future study.

EPVS are conduits for drainage of interstitial fluid from the brain (3,4). In patients with lacunar stroke, EPVS were associated with increased blood–brain barrier (BBB) permeability (24) and the BBB is more permeable in active multiple sclerosis plaques in which EPVS become larger and more numerous during periods of active inflammation (14). Associations between EPVS and inflammatory plasma markers in patients with SVD have also been studied in detail by Rouhl *et al*. including evidence for involvement of activated monocytes/macrophages in cerebral SVD (25). Inflammation may be part of the pathophysiology of both SVD and hypertension so the change in size of EPVS in another disease in which inflammation is known to play an active and specific role (multiple sclerosis) is highly relevant. EPVS may therefore be a marker of BBB dysfunction. EPVS have also been attributed to ex-vacuo dilatation secondary to shrinkage of cerebral tissue after demyelination and axonal loss (26), but this would not explain the disappearance of EPVS from formerly inflamed multiple sclerosis (MS) plaques as the plaques become quiescent – if EPVS are a consequence of atrophy, then in the case of MS, resolution of inflammation with tissue scarring should make the EPVS more visible. In our study, we found an association between atrophy and only BG-EPVS, not total or CS- EPVS. The association with atrophy was not noted in previous studies of patients with ischemic stroke (8) or multiple sclerosis (14). Thus, any association with atrophy may be due to a co-association with age.

Increasingly, EPVS are being recognized as a marker of disease, rather than simply being ignored or considered a marker of ageing; however, data are still relatively scant. Our study provides further evidence that EPVS are another MRI marker for cerebral SVD; however, further investigation of EPVS in studies of ischemic stroke, ageing, and dementia is required to determine their pathophysiological role in these disorders.

## Supporting Information

Table S1 Baseline characteristics of patients undergoing MRI compared with all acute ischemic stroke patients recruited to the stroke register.
