# Supplementary material for: Enlarged perivascular spaces and cerebral small vessel disease
Source: Int J Stroke. 2013 May 22;10(3):376–81. doi: 10.1111/ijs.12054 (PMC4463944; doi:10.1111/ijs.12054)
Supplement: Table S1 — Baseline characteristics of patients undergoing MRI compared with all acute ischemic stroke patients recruited to the stroke register. [file ijs0010-0376-sd1.doc]

**Table S1.** Baseline characteristics of patients undergoing MRI compared to all acute ischemic stroke patients recruited to the stroke register. **Online only**

|  | All acute ischemic stroke patients  n=1311 | Patients with MRI  n=313 |
| --- | --- | --- |
| Age in years (mean ± SD) | 71.4 ± 12.9 | 68.5 ± 13.4 |
| Male sex (%) | 671 (51.2) | 169 (54) |
| Risk factors  Previous stroke | 235 (18.0) | 80 (25.6) |
| Hypertension | 704 (53.7) | 164 (53.1) |
| Diabetes | 166 (12.7) | 28 (9) |
| OCSP classification  TACS  PACS  POCS  LACS  Uncertain | 122 (9.3)  579 (44.2)  207 (15.8)  350 (26.7)  53 (4.0) | 24 (7.7)  136 (43.4)  64 (20.5)  79 (25.2)  10 (3.2) |
